# Evaluation of the small-field output factor in eclipse modeling methods using representative beam and measured data with averaged ionization chamber and diode detector measurements

**DOI:** 10.1007/s13246-025-01676-1

**Published:** 2025-11-24

**Authors:** Kunio Nishioka, Yuki Kunii, Yoshinori Tanabe, Yuichi Sakamoto, Akira Nakamoto, Shotaro Takahashi

**Affiliations:** 1 Department of Radiology, Tokuyama Central Hospital, 1-1 Kodacho, Shunan, Yamaguchi 745-8522 Japan; 2 Department of Radiological Technology, Graduate School of Health Sciences, Okayama University, 5-1 Shikata-cho, 2-chome, Kita-ku, Okayama-shi, 700-8558 Japan

**Keywords:** Beam modeling, Plastic scintillator detector, Small irradiation field, Output factor

## Abstract

Beam modeling for radiotherapy treatment planning systems (RTPS) can be performed using representative beam data (RBD) or direct measurements. However, RBD typically excludes output factor (OPF) measurements for fields smaller than 3 × 3 cm^2^. The Eclipse treatment planning system addresses this limitation by incorporating measured OPF data for fields as small as 1 × 1 cm^2^. Although existing studies have primarily examined the accuracy of small-field OPFs for plastic scintillator detectors, studies directly comparing the OPF values obtained through RBD modeling with and without OPF measurements for small field sizes are limited. Therefore, this study proposes a novel measurement approach using data averaged from an ion chamber and diode detector for small-field dosimetry to provide critical insights into the integration of OPFs for these small field sizes in RBD-based beam modeling. We systematically evaluated the impact of small-field OPF measurements on beam modeling accuracy by comparing three distinct approaches: (1) RBD-based modeling without small-field OPF data, (2) RBD-based modeling incorporating measured small-field OPF data, and (3) modeling based solely on measured data, with and without the inclusion of 1 × 1 cm^2^ field sizes. In addition, we compared OPF values obtained from a W2 plastic scintillator detector with the averaged OPF values from a PinPoint 3D ion chamber and EDGE diode detector across multiple beam energies and flattening filter-free (FFF) configurations. Our analysis included field sizes ranging from 1 × 1 cm^2^ to 40 × 40 cm^2^. The results demonstrated that for square fields, OPF calculation differences between RBD modeling with and without measured data were < 1.5%, < 4.5%, and < 4.5% at 1 × 1 cm^2^, and < 0.5%, < 1.5%, and < 1.5% at 2  ×  2  cm^2^, respectively. The RBD group exhibited a trend in which the OPF difference increased with the expansion of the irradiation field size. Notably, the most significant variations between modeling approaches occurred along the upper jaw expansion direction in rectangular fields. This suggests that a thorough evaluation is necessary for modeling results with an OPF ≤  1 × 1 cm^2^. This study highlights the advantages and disadvantages of beam modeling using measured OPF and RBD, providing valuable insights for future facilities that rely solely on RBD for beam modeling.

## Introduction

Stereotactic radiotherapy and intensity-modulated radiotherapy (IMRT) are extensively utilized in the management of lung cancer, brain metastases, oligo-metastases, and head and neck malignancies [1, 2]. Stereotactic radiotherapy is a technique for treating lung cancer and oligo-metastases by delivering high radiation doses over a limited number of fractions with a small radiation field. Meanwhile, head and neck IMRT employs complex integration of multiple small irradiation fields and elongated rectangular fields. Small or elongated rectangular fields have uncertainties in the accuracy of dose measurements and treatment planning calculations and require rigorous quality control before treatment to ensure consistency between the radiotherapy equipment and the radiotherapy planning system (RTPS) [3, 4]. Achieving precision in dose delivery requires rigorous quality control to ensure consistency between the radiotherapy equipment and the RTPS [3, 4]. Accurate RTPS modeling is crucial for maintaining dose consistency and relies on factors such as the output factor (OPF), multi-leaf collimator (MLC) parameters, and beam profiles [5]. The radiotherapy is delivered using an irradiation field formed by the jaw and MLC. In the RTPS, the OPF formed by the jaw is modeled, and the output formed by both the MLC and jaw is adjusted using MLC parameters. Among these, the OPF is directly associated with the calculated output dose. RTPS modeling is typically commissioned during the installation of linear accelerators and during RTPS version upgrades [6, 7].

In the context of small-field dosimetry for field sizes ≤ 2 × 2 cm^2^, the OPF is subject to uncertainties stemming from factors such as differences in response due to non-tissue-equivalent detector materials, volume averaging of the active detector volume, and lack of electronic equilibrium [8]. Moreover, measurement conditions in small-field settings are often challenging owing to the absence of lateral electronic equilibrium and the influence of an extended focal spot [9]. The IAEA TRS-483 report addresses these complexities by providing correction factors designed for small-field detectors under such conditions [9]. Furthermore, the use of plastic scintillator detectors (PSDs), comprising water-equivalent materials, is recommended for small-field measurements. PSDs with small, water-equivalent sensitive volumes are considered ideal because they do not require correction factors. In addition, the OPF of small irradiation fields can be accurately measured using a PSD or detector with a correction factor [9].

Applying representative beam data (RBD) and golden beam data for beam modeling in RTPS has emerged as an efficient and expedited approach to machine installation, component replacement, and upgrades [10]. This method is adjusted and standardized by the vendor to have identical beam characteristics, thereby eliminating the need for direct measurements of OPF and beam profiles [10]. For example, the RBD of TrueBeam in Eclipse comprises averaged data from three TrueBeam units measured at a single institution using a CC13 ionization chamber (IBA Dosimetry, Schwarzenbruck, Germany) [11]. Therefore, when utilizing RBD, meticulous calibration of jaw positions—relative to field size, beam quality, and dose output—is critical to minimizing discrepancies between the RBD and the actual beam characteristics specific to individual linear accelerator units [11, 12]. The TrueBeam RBD for RTPS Eclipse includes OPF values, percent depth dose (PDD), and off-center ratio (OCR) data for field sizes ≥ 3 × 3 cm^2^. In the Eclipse RTPS, beam modeling using measurement data, such as OPF data for field sizes ≥ 1 × 1 cm^2^ and PDD and OCR data for field sizes ≥ 2 × 2 cm^2^, can be used to configure a treatment model. However, beam scan data of PDD and OCR have limitations in integrating field sizes < 2 × 2 cm^2^. The calculated OPFs in the RTPS after modeling may differ from the measured values because beam modeling incorporates scan data, highlighting the importance of thorough commissioning before clinical implementation.

The IAEA TRS-483 report provides explicit guidelines for deriving output correction factors in rectangular fields using the equivalent square field size methodology [9]. However, for fields with extreme aspect ratios, the accuracy of correction factors and the calculated dose may diminish owing to variations in the dosimeter response and scattering effects [13, 14]. Therefore, dosimetrists must carefully consider the trade-off between beam settings that may compromise dose accuracy and the clinical imperative of minimizing exposure to organs at risk.

For measurements in small irradiation fields (≤ 2 × 2 cm^2^), solid-state detectors typically exhibit over-response owing to their high material density, whereas ionization chambers tend to under-respond owing to their lower density [8, 15, 16]. Output correction factors are provided individually for each detector. For example, for a 1 × 1 cm^2^ field, the output correction factor is 1.004 for the EDGE diode detector (Sun Nuclear Corp., Melbourne, FL) and 0.994 for the PTW 31,016 PinPoint 3D ionization chamber (PTW Freiburg GmbH, Freiburg, Germany), yielding a theoretical average of approximately 0.999. In addition, PSDs are subject to uncertainties related to the relative magnitude of the Cerenkov signal compared with the scintillation signal. These uncertainties arise from beam fiber geometry variations and the directional characteristics of Cerenkov radiation production [17]. Therefore, accurate dose assessments necessitate careful consideration of the respective strengths and limitations of different dosimeter types. Moreover, the correction factors provided for detectors other than the TRS-483 PSD are limited in terms of photon energy, and no correction factor has been reported for the 4 MV photon beam, which is widely used in clinical practice [18, 19]. In this study, we propose a novel approach to PSD evaluation and routine quality assurance by introducing the average OPF derived from both solid-state detectors and ionization chambers. We examine the impact of small-field OPF values through RTPS modeling using RBD—with and without OPF measurements—for field sizes ≤ 2 × 2 cm^2^, incorporating corresponding beam profile data obtained from these measurements.

While existing studies have primarily examined the accuracy of small-field OPFs for PSDs, no existing studies have directly compared OPF values obtained through RBD modeling with and without OPF measurements for field sizes  ≤ 2 ×  2 cm^2^, using the average OPF derived from the PinPoint 3D ionization chamber and EDGE diode detectors. The findings of this study aim to provide critical insights into the integration of OPFs for these small field sizes in RBD-based beam modeling. Considering the limitations of facility-owned detectors, using two distinct response detector types for small-field measurements can reinforce RTPS commissioning procedures and enhance the reliability of routine quality assurance.

## Methods

### Equipment used

This study was conducted using a TrueBeam linear accelerator (Varian Medical Systems, Palo Alto, CA, USA) and the Eclipse 16.1 RTPS (Varian Medical Systems). OPF measurements were performed using the Exradin W2 PSD (Standard Imaging Inc., Middleton, WI), CC13 ionization chamber, PTW 31,016 PinPoint 3D ionization chamber, and EDGE diode detector, in conjunction with a Blue Water Phantom 2 (Standard Imaging Inc., Middleton, WI, USA). Measurements were supported by a MA  × SD electrometer (Standard Imaging Inc.) and a RAMTEC Duo electrometer (Toyo Medic, Tokyo, Japan). Beam alignment was verified and adjusted using an IC Profiler (Sun Nuclear Corp., Melbourne, FL), the CC13 ionization chamber, and the Blue Water Phantom 2. Jaw position adjustments were performed using Gafchromic RTQA2 film (Ashland ISP Inc., Wayne, NJ). The photon beam energies utilized in this study included 4, 6, and 10 MV, as well as 6 and 10 MV flattening filter-free (FFF) beams. Dose calculations were performed using the anisotropic analytical algorithm (AAA) and Acuros XB (AXB).

## Jaw position calibration using graph paper

The jaw position was calibrated because it directly affects the OPF for small irradiation field sizes. Jaw positions at 1 and 19 cm were visually calibrated by projecting the light field onto graph paper placed on the treatment couch at a source-to-surface distance (SSD) of 100 cm. Following visual alignment, a collimator split-field test was conducted using Gafchromic RTQA2 film (ISP, Wayne, NJ) to verify the zero position of the jaw. The irradiation field size, defined as the distance between the 50% dose levels, was fine-tuned to within 1.0 mm for field sizes of 10 × 10, 20 × 20, and 30 × 30 cm^2^ using a 6-MV beam, an SSD of 90 cm, and a depth of 10 cm. The tolerance limits were ± 1 mm for the lower (X) jaws and ± 2 mm for the upper (Y) jaws [20]. These jaw position and field size calibrations were validated by dose profile measurements obtained using the CC13 chamber and the Blue Water Phantom 2. After jaw position calibration, the encoding mechanism of the system ensures a reproducible jaw position accuracy of 0.1 mm [21].

## Beam matching between RBD data and the measured beam quality and profile

Before beam matching, symmetry adjustments were performed for the 4, 6, and 10 MV beams to align the characteristics of beams with and without a flattening filter. These adjustments were conducted in service mode using the IC Profiler before configuring the beam profile settings (PDD and OCR).

The beam quality in this study was evaluated using PDD_10_ and PDD_20_ values, as the RBD data includes PDD information. The beam-quality index, i.e., Tissue–Phantom Ratio_20,10_, was calculated based on the PDD_10_ and PDD_20_ measurements [22]. The beam quality was adjusted by the vendor to within 1% between the RBD and the beam of the facility by comparing PDD values at depths of 10 and 20 cm (PDD_10_, PDD_20_). These measurements were conducted using a 10 × 10 cm^2^ field size at an SSD of 100 cm and at photon energies of 4, 6, 10, 6 MV FFF, and 10 MV FFF. Measurements were performed using the CC13 ionization chamber and Blue Water Phantom 2. In the Blue Phantom 2 setup, automatic central axis correction was performed to ensure that the chamber was properly aligned. Beam symmetry and flatness were evaluated by analyzing the OCR profiles for a 30 × 30 cm^2^ field size at a depth of 10 cm and an SSD of 100 cm. Across all energies, beam quality, symmetry, and flatness were aligned to within a 1% deviation between the RBD and measured data.

## Comparison of OPFs across four types of small-field detectors in square irradiation fields

While the RBD was obtained at a depth of 5 cm and an SSD of 95 cm, this study measured small-field OPFs at a depth of 10 cm and an SSD of 90 cm. This modification was made to minimize uncertainties such as positional deviations during small-field measurements, particularly the 1 cm field. OPFs were measured three times for each field size, using 100 monitor units per measurement, across 11 square field sizes (1 × 1, 2 ×  2, 3 × 3, 4 × 4, 5 × 5, 7  × 7, 10 × 10, 15 × 15, 20 × 20, 30 × 30, and 40 ×  40 cm^2^) at a depth of 10 cm and an SSD of 90 cm. The detectors used included the Exradin W2 PSD (paired with the MAX SD electrometer), CC13 ionization chamber (for field sizes ≥ 3 × 3 cm), PTW 31,016 PinPoint 3D ionization chamber, and EDGE diode detector, all interfaced with the RAMTEC Duo electrometer. The electrometer was operated at −  300 V for the CC13 and PinPoint 3D ionization chambers. OPF measurements obtained using the W2 PSD were compared with those obtained from the three detectors (CC13, PinPoint 3D ionization chamber, and EDGE diode), with the application of TRS483 correction factors. In addition, the average OPF values derived from the PinPoint 3D ionization chamber and EDGE diode detectors were calculated to support further comparative analysis. For example, at 6 MV, the correction factors for the PinPoint 3D ionization chamber and EDGE diode detectors were 1.039 and 0.966 for the 1 × 1 cm^2^ field, 1.004 and 0.994 for the 2 × 2 cm^2^ field, and 1.001 and 0.999 for the 3 × 3 cm^2^ field, respectively. The average of the sums of these correction factors was 1.000 for the 1 ×  1 cm^2^ field, 0.999 for the 2  × 2 cm^2^ field, and 1.000 for the 3 × 3 cm^2^ field. For OPF measurements, each field size was measured at least three times, and the final value was taken as the mean of the sample.

## Daisy-chaining of measured OPFs for beam modeling

Owing to the variable response of small detectors, such as the EDGE diode with increasing field size [8], the daisy-chaining method was employed to ensure consistency in the OPF measurements used for beam modeling [15]. The inconsistencies in the responses between different detector types can be eliminated using the daisy-chaining method [23]. For field sizes ≥ 3 × 3 cm^2^, OPFs were obtained using the CC13 ionization chamber. For smaller field sizes (≤ 2 × 2 cm^2^), the average OPFs measured with the PinPoint 3D ionization chamber and EDGE diode were used. These small-field OPFs were subsequently renormalized to align with the CC13 ionization chamber measurement at the 4 × 4 cm^2^ field size using the intermediate field method, also known as daisy-chaining (Table 1). For each OPF measurement, a minimum of three repeated measurements were performed per field size, and the mean value was used. A coefficient of variation of ≤ 0.1 was adopted as the tolerance threshold. The relative standard uncertainty of detectors was calculated for each field size to understand the uncertainty of measurements in small-field measurements.

### Measurement of PDD and cross-plane profiles ≥  2 × 2 cm^2^ for beam modeling

PDD measurements were performed for eight square field sizes (2  × 2, 3 × 3, 4 × 4, 6 × 6, 10 ×  10, 20 × 20, 30 × 30, and 40  × 40 cm^2^) at an SSD of 100 cm using the CC13 ionization chamber and the Blue Water Phantom 2.

Cross-plane dose profiles for the same field sizes were measured at five depths—dose maximum, 5 cm, 10 cm, 20 cm, and 30 cm—using the CC13 ionization chamber and the Blue Water Phantom 2.

## Three types of beam modeling using RBD and measurement data

Three distinct beam modeling groups were configured in the RTPS, based on different combinations of RBD and measurement data: the RBD, M, and RBD + M groups, each representing a configuration that may be introduced into clinical practice.

The RBD group included beam profile data (PDD and cross-plane profile) and OPFs for field sizes  ≥ 3 × 3 cm^2^, all obtained from RBD (Table 1). The M group included measured beam profile data for field sizes  ≥ 2 × 2 cm^2^ and measured OPFs for field sizes ≥ 1 × 1 cm^2^ (using average OPF values calculated from the PinPoint 3D ionization chamber and EDGE diode for field sizes of 1  ×  1 and 2 × 2 cm^2^, and for the CC13 ionization chamber: ≥ 3 ×  3 cm^2^; Table 1). The RBD+M group combined beam profile data from the RBD group for field sizes ≥ 3 × 3 cm^2^ with OPFs for field sizes ≥ 1 × 1 cm^2^ (using average OPF values calculated from the PinPoint 3D ionization chamber and EDGE diode for field sizes of 1 × 1 and 2 × 2 cm^2^, and for the CC13 ionization chamber: ≥ 3  ×  3 cm^2^; Table 1).

## Comparison between the three modeling types using the calculated and measured OPFs

The OPFs were calculated using the AAA and AXB algorithms for all three beam modeling groups—RBD, M, and RBD +M—for square and rectangular irradiation fields. These calculations included all combinations of upper and lower jaw positions (field widths: 1–5, 7, 10, 15, 20, 30, and 40 cm) at a depth of 10 cm and an SSD of 90 cm, using a 1 mm dose calculation grid in the RTPS.

The Kruskal–Wallis test was employed to evaluate the statistical significance of OPF differences among the three modeling groups across various beam energies. Calculated OPFs from each modeling group were compared against measured OPFs from the Exradin W2 PSD (for square fields  ≤ 2 × 2 cm^2^, rectangular fields with one side measuring ≤ 1–2 cm) and from the CC13 ionization chamber (for fields ≥ 3 × 3 cm^2^). In addition, rectangular field OPFs (1 × 2, 1  ×  3, 1 × 4, 1 × 5, 1  ×  7, 1  ×  10, 1 × 15, 1  × 20, 1 × 30, and 1  ×  40 cm^2^) were analyzed by fixing one jaw (either upper or lower) to a 1 cm width, thereby evaluating the dose calculation algorithm under asymmetric field conditions.

### Statistical analysis

All statistical analyses were conducted using SPSS version 29.0 (IBM Corporation, Armonk, NY, USA). The Kruskal–Wallis test with Bonferroni correction was used to assess significance, with a* p*-value < 0.01 considered statistically significant.

**Table 1:** Beam modeling data used across the three study groups

Group	Field size (cm^2^)	Scan profile data	Non-scan data		
			OPF	Detector	Intermediate field as daisy-chaining
RBD	1 × 1	–	–	–	–
	2 × 2	–	–	–	
	≥ 3 × 3	RBD	RBD	CC13	
M	1 × 1	–	Measurement	PinPoint 3D and EDGE	4 × 4 cm^2^
	2 × 2	Measurement			
	≥ 3 × 3	Measurement	Measurement	CC13	
RBD + M	1 × 1	–	Measurement	PinPoint 3D and EDGE	4 × 4 cm^2^
	2 × 2	–			
	≥ 3 × 3	RBD	Measurement	CC13	

## Results

**Fig. 1:**
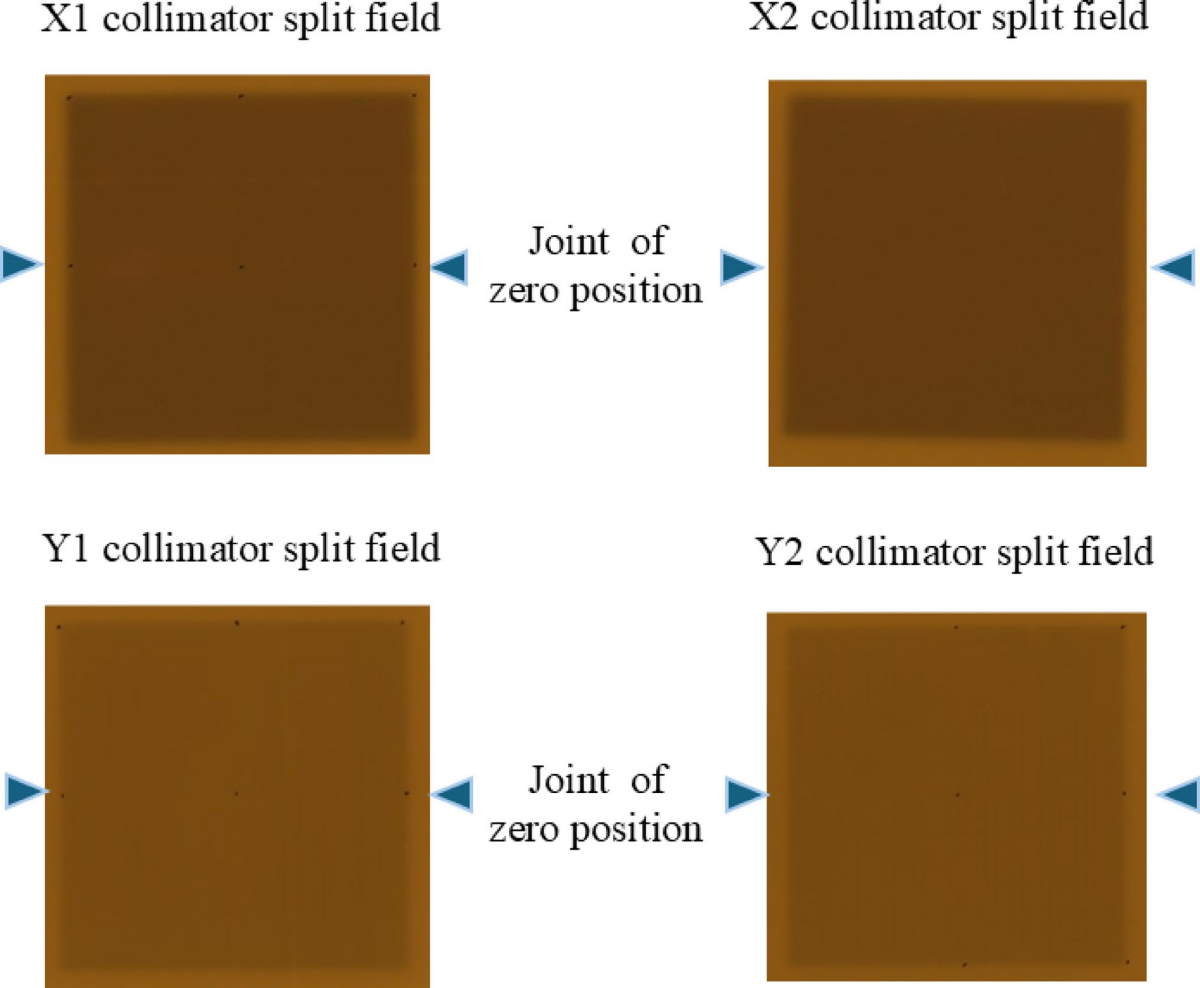
Collimator split-field test using RTQA2 film and measurement of irradiation field size with a 3D water phantom

The results of the collimator split-field test and irradiation field size measurements for the 6 MV beam are shown in Fig. 1. In the split-field test, inverting the collimator and aligning the jaws at the zero position yielded no observable over- or under-dosing at the isocenter, confirming accurate jaw alignment. For field sizes of 10  × 10, 20 ×  20, and 30 ×  30 cm^2^, the deviations in the measured field dimensions were within 1.0 mm, demonstrating a high level of precision in jaw positioning and field size calibration (Table 2).

**Table 2:** Measurement field sizes for each jaw following jaw position calibration

Field size (cm)		Measurement field size (cm)	Difference (cm)
10.0	X jaw	10.07	0.07
	Y jaw	10.03	0.03
20.0	X jaw	20.00	0.00
	Y jaw	19.97	− 0.03
30.0	X jaw	29.96	− 0.04
	Y jaw	29.98	− 0.02

The measured PDD_10_ and TPR_20,10_ values were adjusted to ensure deviations of less than 1% compared with the values obtained from the RBD, as shown in Table 3. For all beam energies, the reference field size of 10  × 10 cm^2^ showed agreement within ±  0.2% for both PDD_10_ and TPR_20,10_. Deviations exceeding 0.5% were observed for PDD_10_ in the 30  ×  30 cm^2^ field at 6 MV and for TPR_20,10_ in the 3 ×  3 cm^2^ and 30 × 30 cm^2^ fields at 4 MV.

**Table 3:** Dosimetric adjustments between RBD and facility-specific beam parameters

Energy	Field size (cm^2^)	PDD_10_			TPR_20,10_		
		RBD	Measured	Difference (%)	RBD	Measured	Difference (%)
4 MV	3.0 × 3.0	0.550	0.550	0.00	0.569	0.572	0.52
	4.0 × 4.0	0.564	0.564	0.00	0.578	0.578	0.00
	10.0 × 10.0	0.625	0.624	− 0.16	0.622	0.621	− 0.16
	30.0 × 30.0	0.685	0.682	− 0.44	0.703	0.699	− 0.57
6 MV	3.0 × 3.0	0.605	0.603	− 0.33	0.620	0.622	0.32
	4.0 × 4.0	0.615	0.615	0.00	0.627	0.629	0.32
	10.0 × 10.0	0.664	0.663	− 0.15	0.667	0.668	0.15
	30.0 × 30.0	0.710	0.706	− 0.57	0.735	0.735	0.00
10 MV	3.0 × 3.0	0.699	0.699	0.00	0.702	0.704	0.28
	4.0 × 4.0	0.707	0.707	0.00	0.707	0.708	0.14
	10.0 × 10.0	0.735	0.736	0.14	0.740	0.739	− 0.14
	30.0 × 30.0	0.759	0.757	− 0.26	0.788	0.787	− 0.13
6 MV FFF	3.0 × 3.0	0.570	0.569	− 0.18	0.589	0.588	− 0.17
	4.0 × 4.0	0.584	0.582	− 0.34	0.594	0.595	0.17
	10.0 × 10.0	0.635	0.633	− 0.32	0.633	0.632	− 0.16
	30.0 × 30.0	0.677	0.674	− 0.45	0.688	0.686	− 0.29
10 MV FFF	3.0 × 3.0	0.668	0.669	0.15	0.674	0.674	0.00
	4.0 × 4.0	0.679	0.679	0.00	0.678	0.680	0.29
	10.0 × 10.0	0.711	0.711	0.00	0.708	0.708	0.00
	30.0 × 30.0	0.730	0.729	− 0.14	0.741	0.742	0.13

**Fig. 2:**
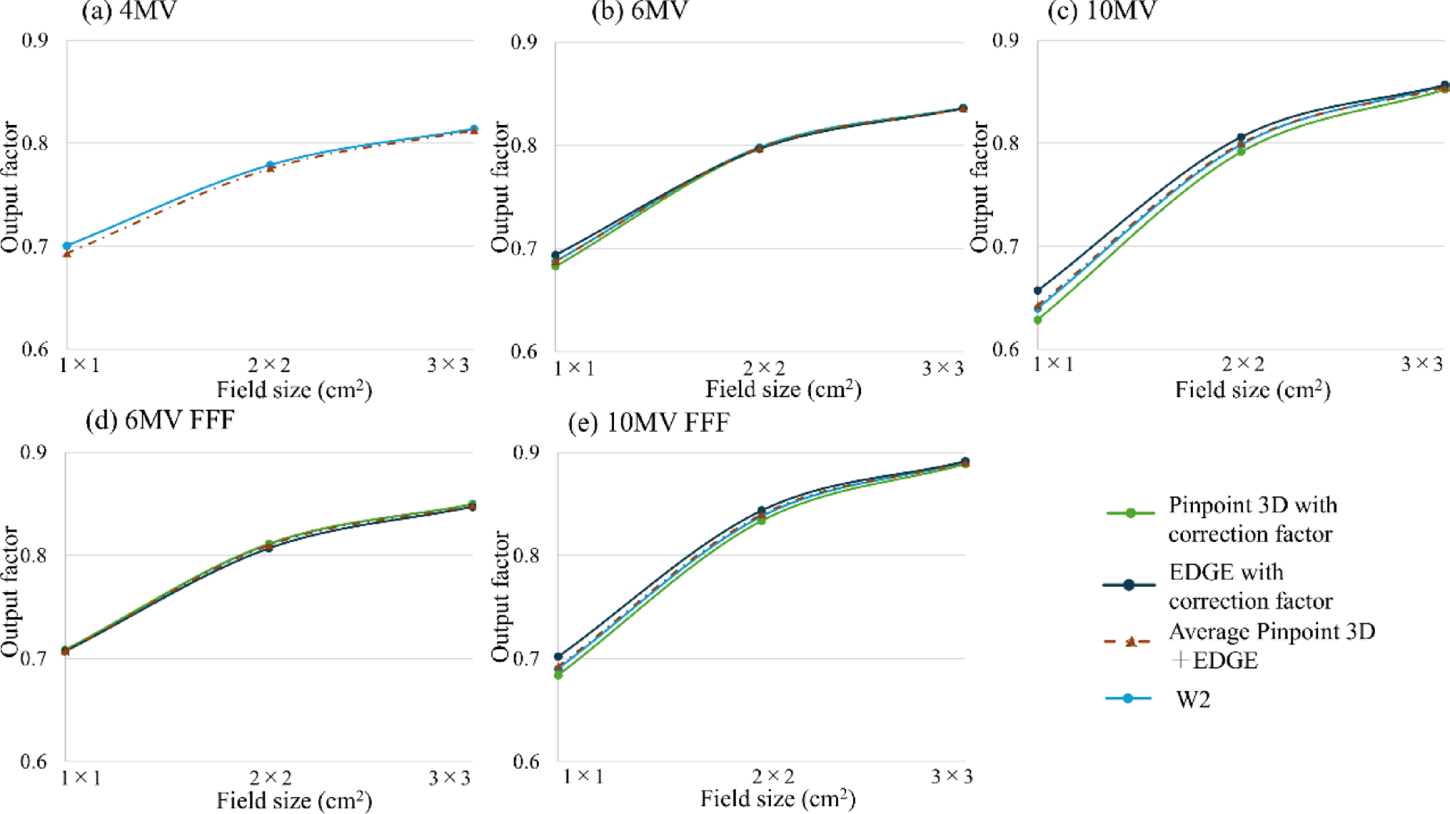
Relationship between the OPF and detector response at** a** 4 MV,** b** 6 MV,** c** 10 MV,** d** 6 MV FFF, and** e** 10 MV FFF

The relationship between the OPF and small-field detectors, including the PinPoint 3D ionization chamber and EDGE diode with and without correction factors, is presented in Fig. 2; Table 4. When comparing OPFs measured with the W2 PSD (≤ 2  × 2 cm^2^) and the CC13 ionization chamber (≥ 3 × 3 cm^2^), differences of over 2% and less than 1.2% were observed, respectively. (Fig. 2; Table 4).

The average OPF derived from the PinPoint 3D ionization chamber and EDGE diode showed <  1.0% without a correction factor (Table 4).

**Table 4:** Differences in the OPFs between the W2 PSD and other chambers

Difference (%)	Field size (cm^2^)	Base		With the correction factor						Average
		W2		CC13		PinPoint 3D		EDGE		EDGE and PinPoint 3D
		Diff	SD	Diff	SD	Diff	SD	Diff	SD	Diff
4 MV	1 × 1	–	0.001	–		–	–	–	–	–1.06
	2 × 2	–	0.001	–	–	–	–	–	–	− 0.45
	3 × 3	–	0.000	–	–	–	–	–	–	− 0.18
	4 × 4	–	0.001	–	–	–	–	–	–	0.00
	5 × 5	–	0.001	–	–	–	–	–	–	− 0.03
6 MV	1 × 1	–	0.001	–	0.001	− 0.69	0.085	0.89	0.006	0.01
	2 × 2	–	0.001	− 0.32	0.001	− 0.18	0.030	− 0.21	0.009	− 0.09
	3 × 3	–	0.001	− 0.10	0.001	− 0.07	0.096	− 0.11	0.007	− 0.09
	4 × 4	–	0.001	0.00	0.001	0.00	0.029	0.00	0.012	0.00
	5 × 5	–	0.001	0.04	0.001	0.05	0.042	0.03	0.020	0.04
10 MV	1 × 1	–	0.001	–	0.001	− 1.72	0.055	2.71	0.014	0.46
	2 × 2	–	0.001	− 1.58	0.001	− 0.85	0.038	0.96	0.008	0.16
	3 × 3	–	0.001	− 0.52	0.001	− 0.47	0.072	0.07	0.010	− 0.20
	4 × 4	–	0.001	0.00	0.001	0.00	0.065	0.00	0.007	0.00
	5 × 5	–	0.001	0.09	0.001	0.03	0.071	–0.04	0.006	− 0.01
6 MV FFF	1 × 1	–	0.000	–		0.04	0.010	–0.12	0.007	− 0.16
	2 × 2	–	0.000	− 0.02	0.001	0.03	0.060	− 0.50	0.019	− 0.13
	3 × 3	−	0.001	− 0.07	0.001	− 0.03	0.040	− 0.32	0.005	− 0.17
	4 × 4	–	0.002	0.00	0.001	0.00	0.046	0.00	0.009	0.00
	5 × 5	–	0.000	0.10	0.001	0.09	0.052	0.27	0.011	0.18
10 MV FFF	1 × 1	–	0.001	–		− 0.93	0.125	1.69	0.010	0.31
	2 × 2	–	0.001	− 0.93	0.001	− 0.53	0.212	0.69	0.011	0.18
	3 × 3	–	0.002	− 0.24	0.000	− 0.25	0.032	0.05	0.015	− 0.10
	4 × 4	–	0.001	0.00	0.002	0.00	0.214	0.00	0.013	0.00
	5 × 5	–	0.001	0.03	0.001	0.02	0.066	− 0.06	0.012	− 0.02

The relative standard uncertainties of the W2 PSD, PinPoint 3D ionization chamber, and EDGE diode for OPF measurement were 0.00, < 0.02, and <  0.09% for irradiation fields ≤  4  × 4 cm^2^, across all energies (Table 5).

**Table 5:** The relative standard uncertainty of OPF measurement for irradiation field  ≤  4 × 4 cm^2^ include rectangular fields

Relative standard uncertainty (%)		One side field size (cm)			
		1	2	3	4
W2 PSD	Upper jaw	0.001	0.001	0.001	0.001
	Lower jaw	0.001	0.001	0.001	0.001
EDGE	Upper jaw	0.011	0.010	0.012	0.011
	Lower jaw	0.011	0.011	0.011	0.011
Pinpoint3D	Upper jaw	0.069	0.058	0.065	0.060
	Lower jaw	0.070	0.077	0.073	0.088

The variations in the OPFs measured by the W2 PSD (≤ 2  × 2 cm^2^) and CC13 ionization chamber (≥ 3 × 3 cm^2^) across the three beam modeling groups for small-field calculations are summarized in Table 6. For the 1 × 1 cm^2^ irradiation field, the differences between the W2 PSD-measured and average OPFs across all photon energies for the AXB and AAA algorithms were as follows: RBD group = −  1.78%, −  0.78%; M group = −  3.95%, −  2.41%; and RBD +M group = −  3.93%, −  2.40%, respectively. The standard deviations of OPFs across different energies for the 1 ×  1 cm^2^ field were RBD group =  1.05, 0.93; M group = 0.34, 0.39; and RBD+M group  =  0.32, 0.41 for AXB and AAA, respectively.

For the 1 × 3 cm^2^ rectangular field (upper jaw side), the absolute average OPF differences across all energies for the three modeling groups were AXB = 1.44%, 1.62%, and 1.67%; AAA = 1.98%, 1.04%, and 1.03%, respectively. For the 3 × 1 cm^2^ rectangular field (lower jaw side), the absolute average OPF differences were AXB = 0.98%, 3.40%, and 3.48%; AAA = 0.54%, 2.28%, and 2.32%, respectively.

**Table 6:** Differences in OPF for small irradiation fields among the modeling methods, compared with the W2 PSD (≤ 2 × 2 cm^2^) and CC13 ionization chamber (≥ 3 × 3 cm^2^) measurements; differences exceeding 2% are highlighted in Bold

Difference (%)				AcurosXB					AAA				
				Lower jaw					Lower jaw				
				1 cm	2 cm	3 cm	4 cm	5 cm	1 cm	2 cm	3 cm	4 cm	5 cm
RBD group	4 MV	Upper jaw	1 cm	− 0.47	− 0.67	− 0.67	− 0.59	− 0.75	− 1.57	− 1.17	− 0.99	− 0.72	− 0.70
			2 cm	0.41	0.37	0.32	0.33	0.30	− 1.21	− 0.12	0.01	0.19	0.17
			3 cm	− 0.47	− 0.02	− 0.06	− 0.16	− 0.36	− 1.51	− 0.50	− 0.20	− 0.13	− 0.17
			4 cm	− 0.23	0.14	− 0.12	− 0.31	− 0.49	− 1.27	− 0.33	− 0.25	− 0.13	− 0.31
			5 cm	− 0.20	0.12	− 0.21	− 0.42	− 0.50	− 1.23	− 0.19	− 0.18	− 0.09	− 0.17
	6 MV	Upper jaw	1 cm	0.69	0.73	0.73	0.74	0.74	0.69	0.73	0.74	0.75	0.75
			2 cm	0.74	0.80	0.82	0.83	0.83	0.73	0.79	0.81	0.82	0.83
			3 cm	0.75	0.82	0.84	0.85	0.86	0.74	0.81	0.83	0.85	0.86
			4 cm	0.75	0.83	0.85	0.87	0.88	0.75	0.82	0.85	0.87	0.88
			5 cm	0.76	0.84	0.86	0.88	0.89	0.75	0.83	0.86	0.88	0.90
	10 MV	Upper jaw	1 cm	**− 2.66**	**− 2.14**	**− 2.03**	**− 2.04**	**− 2.05**	− 1.48	− 0.58	− 0.17	− 0.20	− 0.22
			2 cm	− 1.52	− 0.41	− 0.41	− 0.35	− 0.30	**− 2.41**	− 1.24	− 0.65	− 0.59	− 0.54
			3 cm	**− 2.33**	− 0.65	− 0.22	− 0.47	− 0.39	**− 2.41**	− 1.17	− 0.19	− 0.31	− 0.24
			4 cm	**− 2.23**	− 0.54	− 0.23	− 0.38	− 0.46	**− 2.47**	− 1.06	− 0.07	− 0.09	− 0.04
			5 cm	**− 2.21**	− 0.51	− 0.35	− 0.38	− 0.54	**− 2.29**	− 1.03	− 0.19	− 0.10	− 0.13
	6 MV FFF	Upper Jaw	1 cm	− 0.69	− 0.39	− 0.41	− 0.23	− 0.20	0.32	0.93	1.26	1.42	1.63
			2 cm	− 0.73	0.06	0.04	0.07	0.41	− 1.57	− 0.57	− 0.26	− 0.06	0.11
			3 cm	− 1.72	− 0.21	− 0.04	− 0.17	− 0.20	− 1.86	− 0.67	− 0.18	− 0.14	− 0.18
			4 cm	− 1.84	− 0.37	− 0.33	− 0.51	− 0.50	− 1.80	− 0.66	− 0.31	− 0.19	− 0.18
			5 cm	− 1.93	− 0.51	− 0.36	− 0.57	− 0.64	− 1.89	− 0.81	− 0.34	− 0.10	− 0.18
	10 MV FFF	Upper jaw	1 cm	**− 2.66**	**− 2.14**	**− 2.14**	**− 2.04**	**− 2.05**	− 1.48	− 0.58	− 0.17	− 0.20	− 0.22
			2 cm	− 1.52	− 0.41	− 0.41	− 0.35	− 0.30	**− 2.41**	− 1.24	− 0.65	− 0.59	− 0.54
			3 cm	**− 2.33**	− 0.65	− 0.22	− 0.47	− 0.39	**− 2.41**	− 1.17	− 0.19	− 0.31	− 0.24
			4 cm	**− 2.23**	− 0.54	− 0.23	− 0.38	− 0.46	**− 2.47**	− 1.06	− 0.07	− 0.09	− 0.04
			5 cm	**− 2.21**	− 0.51	− 0.35	− 0.38	− 0.54	**− 2.29**	− 1.03	− 0.19	− 0.10	− 0.13
M group	4 MV	Upper jaw	1 cm	**− 4.48**	**− 3.93**	**− 3.72**	**− 3.78**	**− 3.74**	**− 3.11**	**− 2.62**	**− 2.41**	**− 2.49**	**− 3.11**
			2 cm	− 1.51	− 0.18	− 0.21	− 0.18	− 0.02	− 1.32	− 1.06	− 0.90	− 0.86	− 1.32
			3 cm	**− 2.17**	− 0.89	− 0.39	− 0.63	− 0.49	− 1.62	− 1.06	− 0.05	− 0.13	− 1.62
			4 cm	**− 1.92**	− 0.87	− 0.42	− 0.60	− 0.44	− 1.37	− 0.87	0.07	0.05	− 1.37
			5 cm	**− 2.05**	− 0.71	− 0.34	− 0.53	− 0.28	− 1.50	− 0.88	− 0.01	− 0.06	− 1.50
	6 MV	Upper jaw	1 cm	**− 3.61**	**− 3.34**	**− 3.26**	**− 3.38**	**− 3.29**	**− 2.16**	**− 2.32**	**− 2.07**	**− 2.20**	**− 2.29**
			2 cm	− 0.80	0.25	0.32	0.21	0.08	− 0.85	− 0.75	− 0.66	− 0.61	− 0.72
			3 cm	− 1.23	− 0.23	− 0.26	− 0.27	− 0.11	− 0.76	− 0.74	0.02	0.01	0.02
			4 cm	− 1.36	− 0.20	− 0.18	− 0.27	− 0.10	− 0.89	− 0.54	0.10	0.16	0.18
			5 cm	− 1.28	− 0.17	− 0.14	− 0.24	− 0.05	− 0.98	− 0.51	− 0.01	0.04	0.08
	10 MV	Upper jaw	1 cm	**− 3.90**	**− 3.40**	**− 3.25**	**− 3.25**	**− 3.09**	**− 2.29**	− 1.91	− 1.79	− 1.96	− 1.81
			2 cm	− 1.12	0.18	0.04	0.10	0.15	− 0.63	− 0.56	− 0.53	− 0.46	− 0.41
			3 cm	− 1.61	− 0.48	− 0.32	− 0.55	− 0.34	− 0.97	− 0.63	− 0.04	− 0.14	− 0.06
			4 cm	− 1.67	− 0.51	− 0.31	− 0.44	− 0.25	− 0.88	− 0.65	0.10	0.09	0.01
			5 cm	− 1.64	− 0.48	− 0.15	− 0.18	− 0.19	− 1.17	− 0.62	− 0.02	0.09	− 0.06
	6 MV FFF	Upper jaw	1 cm	**− 3.80**	**− 3.70**	**− 3.49**	**− 3.48**	**− 3.25**	**− 2.32**	**− 2.28**	**− 2.09**	**− 2.08**	**− 2.04**
			2 cm	− 1.26	− 0.23	− 0.07	− 0.03	0.31	− 0.77	− 0.77	− 0.59	− 0.55	− 0.37
			3 cm	− 1.72	− 0.65	− 0.46	− 0.41	− 0.44	− 0.88	− 0.68	0.00	0.04	0.01
			4 cm	− 1.83	− 0.63	− 0.42	− 0.44	− 0.41	− 1.00	− 0.66	0.04	0.01	0.03
			5 cm	− 1.92	− 0.77	− 0.29	− 0.33	− 0.25	− 1.26	− 0.80	0.00	0.11	0.04
	10 MV FFF	Upper jaw	1 cm	**− 3.90**	**− 3.40**	**− 3.25**	**− 3.25**	**− 3.09**	**− 2.29**	− 1.91	− 1.79	− 1.96	− 1.81
			2 cm	− 1.12	0.18	0.04	0.10	0.15	− 0.63	− 0.56	− 0.53	− 0.46	− 0.41
			3 cm	− 1.61	− 0.48	− 0.32	− 0.55	− 0.34	− 0.97	− 0.63	− 0.04	− 0.14	− 0.06
			4 cm	− 1.67	− 0.51	− 0.31	− 0.44	− 0.25	− 0.88	− 0.65	0.10	0.09	0.01
			5 cm	− 1.64	− 0.48	− 0.15	− 0.18	− 0.19	− 1.17	− 0.62	− 0.02	0.09	− 0.06
RBD + M group	4 MV	Upper Jaw	1 cm	**− 4.48**	**− 3.93**	**− 3.72**	**− 3.78**	**− 4.48**	**− 3.11**	**− 2.80**	**− 2.41**	**− 2.67**	**− 2.45**
			2 cm	− 1.51	− 0.18	− 0.21	− 0.18	− 1.51	− 1.32	− 1.06	− 0.90	− 0.86	− 0.70
			3 cm	**− 2.17**	− 0.89	− 0.39	− 0.63	− 2.17	− 1.62	− 1.06	− 0.05	− 0.13	0.00
			4 cm	− 1.92	− 0.87	− 0.42	− 0.60	− 1.92	− 1.37	− 0.87	0.07	0.05	0.04
			5 cm	**− 2.05**	− 0.71	− 0.34	− 0.53	− 2.05	− 1.50	− 0.88	− 0.01	− 0.06	0.03
	6 MV	Upper jaw	1 cm	**− 3.61**	**− 3.34**	**− 3.26**	**− 3.38**	**− 3.29**	**− 2.16**	**− 2.32**	**− 2.07**	**− 2.20**	**− 2.29**
			2 cm	− 0.80	0.25	0.32	0.21	0.08	− 0.85	− 0.75	− 0.66	− 0.61	− 0.72
			3 cm	− 1.23	− 0.23	− 0.26	− 0.27	− 0.11	− 0.76	− 0.74	0.02	0.01	0.02
			4 cm	− 1.36	− 0.20	− 0.18	− 0.27	− 0.10	− 0.89	− 0.54	0.10	0.16	0.18
			5 cm	− 1.28	− 0.17	− 0.14	− 0.24	− 0.05	− 0.98	− 0.51	− 0.01	0.04	0.08
	10 MV	Upper jaw	1 cm	**− 3.90**	**− 3.40**	**− 3.25**	**− 3.25**	**− 3.26**	**− 2.29**	**− 1.91**	− 1.79	− 1.96	− 1.81
			2 cm	− 1.12	0.18	0.04	0.10	0.15	− 0.63	− 0.56	− 0.53	− 0.46	− 0.41
			3 cm	− 1.61	− 0.48	− 0.32	− 0.55	− 0.34	− 0.97	− 0.63	− 0.04	− 0.14	− 0.06
			4 cm	− 1.67	− 0.51	− 0.31	− 0.44	− 0.25	− 0.88	− 0.65	0.10	0.09	0.01
			5 cm	− 1.64	− 0.48	− 0.15	− 0.18	− 0.19	− 1.17	− 0.62	− 0.02	0.09	− 0.06
	6 MV FFF	Upper jaw	1 cm	**− 3.74**	**− 3.46**	**− 3.26**	**− 3.25**	**− 3.20**	**− 2.32**	**− 2.28**	**− 2.09**	**− 2.08**	**− 2.04**
			2 cm	− 1.04	− 0.03	− 0.04	− 0.01	0.33	− 0.77	− 0.77	− 0.59	− 0.55	− 0.21
			3 cm	− 1.50	−0.62	− 0.43	− 0.39	− 0.42	− 0.88	− 0.68	0.00	0.04	0.01
			4 cm	− 1.62	− 0.61	− 0.39	− 0.57	− 0.40	− 1.00	− 0.66	0.04	0.01	0.03
			5 cm	− 1.88	− 0.75	− 0.27	− 0.47	− 0.24	− 1.26	− 0.80	0.00	− 0.05	0.04
	10 MV FFF	Upper jaw	1 cm	**− 3.90**	**− 3.40**	**− 3.25**	**− 3.25**	**− 3.26**	**− 2.29**	− 1.91	− 1.79	− 1.96	− 1.81
			2 cm	− 1.12	0.18	0.04	0.10	0.15	− 0.63	− 0.56	− 0.53	− 0.46	− 0.41
			3 cm	− 1.61	− 0.48	− 0.32	− 0.55	− 0.34	− 0.97	− 0.63	− 0.04	− 0.14	− 0.06
			4 cm	− 1.67	− 0.51	− 0.31	− 0.44	− 0.25	− 0.88	− 0.65	0.10	0.09	0.01
			5 cm	− 1.64	− 0.48	− 0.15	− 0.18	− 0.19	− 1.17	− 0.62	− 0.02	0.09	− 0.06

**Fig. 3:**
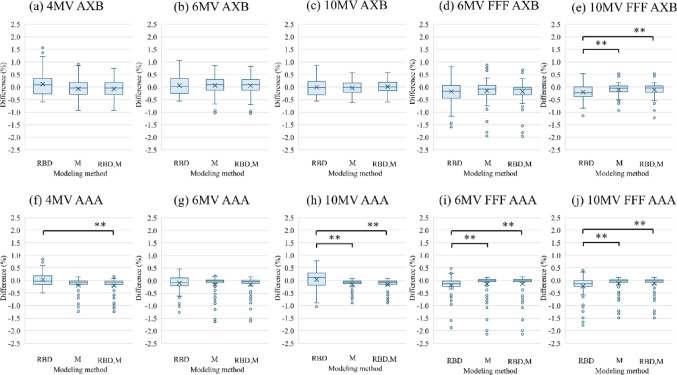
OPF differences among the three modeling methods for field sizes ranging from 2 × 2 to 40  × 40 cm^2^, including rectangular fields:** a** 4 MV AXB;** b** 6 MV AXB;** c** 10 MV AXB;** d** 6 MV FFF AXB;** e** 10 MV FFF AXB;** f** 4 MV AAA (RBD vs. RBD + M);** g** 6 MV AAA;** h** 10 MV AAA (RBD vs. M and RBD + M);** i** 6 MV FFF AAA; and** j** 10 MV FFF AAA. Statistical significance was determined using the nonparametric Kruskal–Wallis test with Bonferroni correction (**: *p* < 0.01)

The box plots in Fig. 3 show the OPF differences for field sizes ranging from 2  ×  2 cm^2^ to 40 ×  40 cm^2^, including rectangular fields, across the three modeling methods. These differences were evaluated by comparing OPFs measured with the W2 PSD (≤ 2  × 2 cm^2^) and CC13 ionization chamber (≥  3  ×  3 cm^2^). For OPFs ≥  2 cm^2^, the differences were within ±  2.5%, with a median deviation of less than ± 0.3% across all energy levels. The differences between the RBD group and the M or M+RBD group tended to be larger for AXB than for AAA, exceeding 1% for 30  ×  40 cm^2^ and 40 ×  40 cm^2^ field sizes at 4 MV; however, they were within 1% for all other energies and algorithms. The largest OPF difference was −  2.14%, observed in the 2  ×  40 cm^2^ rectangular field (aspect ratio: 20) of the RBD+M group at 6 MV FFF using the AAA algorithm. No statistically significant OPF differences were observed between the M and RBD+M groups at any energy level. However, the RBD group exhibited statistically significant differences compared with the RBD+ M group at 4 MV AAA and the M and RBD +M groups at 10 MV AAA, 6 MV FFF, and 10 MV FFF (*p* < 0.01). For AXB, the RBD group also exhibited a statistically significant difference compared with the M and RBD + M groups at 10 MV FFF (*p* <  0.01).

**Fig. 4:**
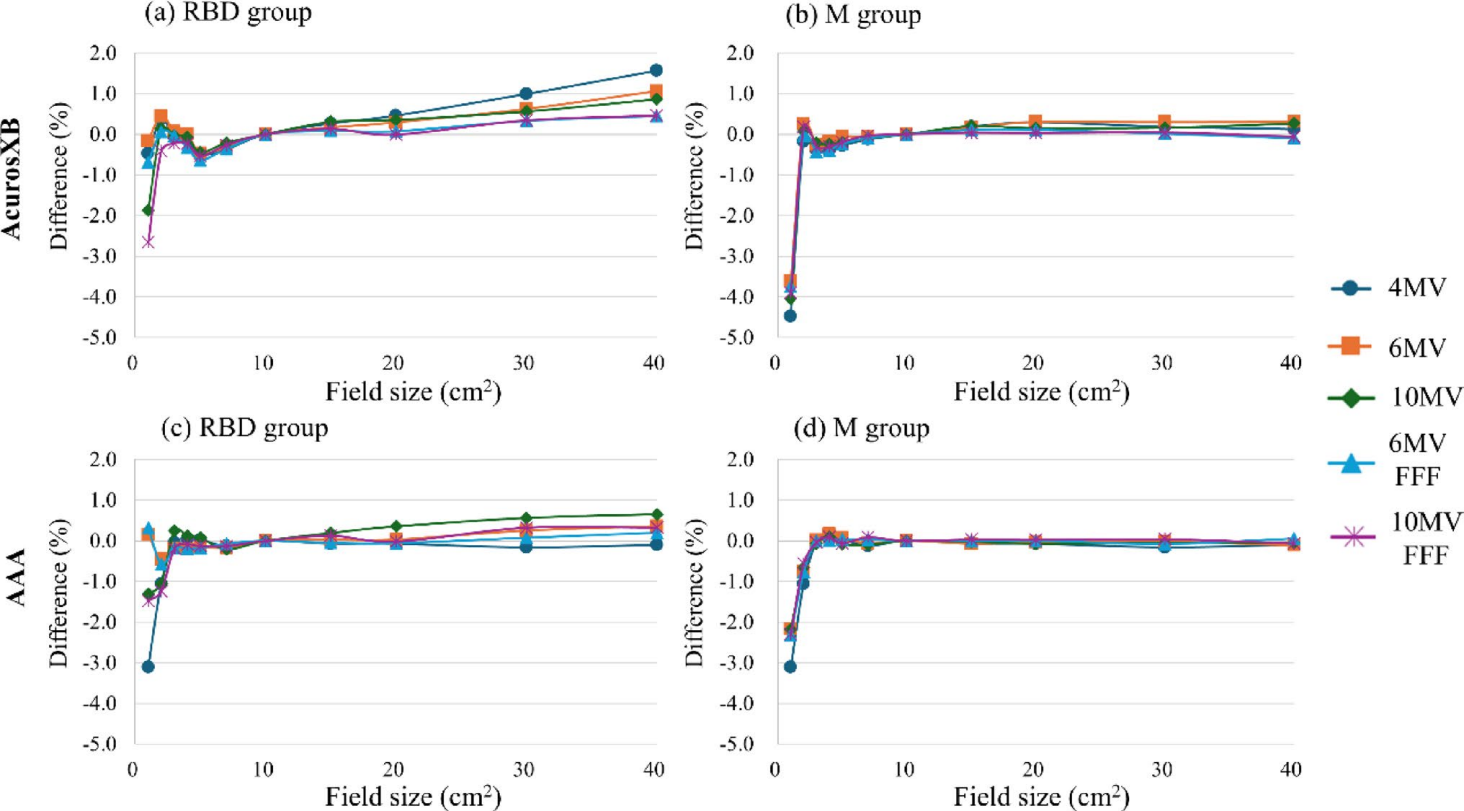
Relationship between the OPF difference and square field size among the modeling methods, compared with the W2 PSD (≤ 2 × 2 cm^2^) and CC13 ionization chamber (≥ 3 × 3 cm^2^) measurements

**Fig. 5:**
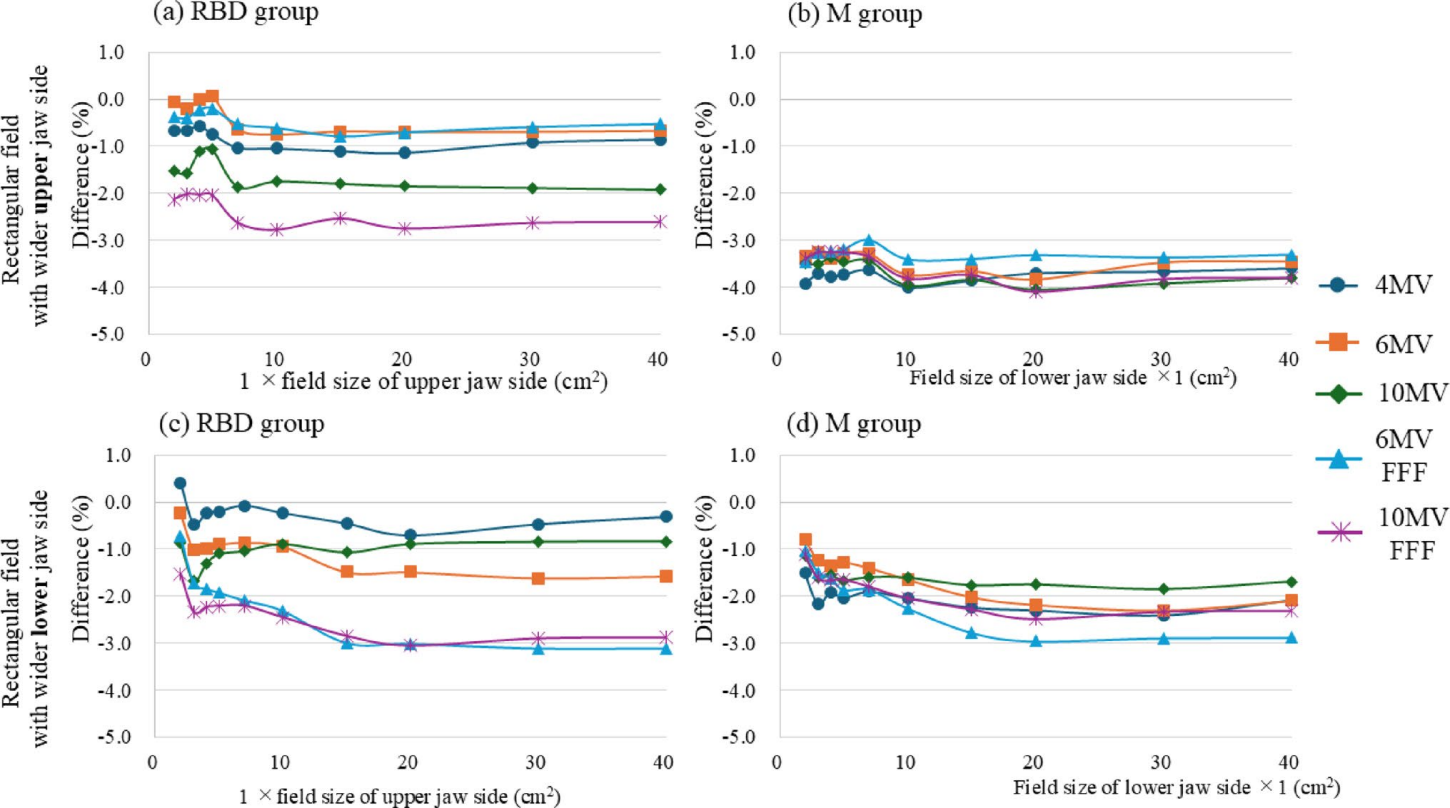
Relationship between the OPF difference and rectangular field size among the modeling methods using AXB, compared with the W2 PSD (≤ 2 × 2 cm^2^) and CC13 ionization chamber (≥ 3 × 3 cm^2^) measurements

Figures 4 and 5 show the relationship between field size and the differences in the OPFs among the three beam modeling groups for square and rectangular fields (with upper and lower jaw configurations). Here, the M and RBD + M groups were not compared because the OPF showed similar results. For square fields, the RBD group exhibited a trend in which the OPF difference increased with the expansion of the irradiation field size, in contrast to the M and RBD + M groups. In addition, the RBD group demonstrated greater variability in OPF differences across beam energies for the square and rectangular fields (Figs. 4 and 5). Notably, in the case of rectangular fields, energy-dependent variations in AXB were more pronounced along the expansion axis of the lower jaw compared with the upper jaw.

The difference in the OPFs for rectangular fields with a fixed lower jaw was greater than that for fields with a fixed upper jaw. This observation was consistent among the M and RBD groups and across the AXB and AAA dose calculation algorithms.

## Discussion

This study evaluated the effectiveness of an averaging method utilizing the PinPoint 3D ionization chamber and EDGE diode. The differences in beam modeling using RBD data with and without incorporating measured OPFs for small irradiation fields, including rectangular configurations, were examined. When evaluating OPFs for small fields, the deviation between the W2 PSD measurements and the average method was generally less than 0.5%, except for a 1.06% discrepancy at 1 × 1 cm^2^ and 4 MV. This deviation can be attributed to the average TRS-483 correction factor for the PinPoint 3D ionization chamber and EDGE diode, which is approximately 1.00 for field sizes of 1 × 1, 2 × 2, and 3 × 3 cm^2^. These findings demonstrate strong agreement with W2 PSD measurements [9]. Studies have shown that OPFs ≤ 2 × 2 cm^2^ exhibit individual variations even when using the same type of dosimeter; however, applying appropriate correction factors can limit these differences to within ≤ 2% for OPFs ≤ 2 × 2 cm^2^ [9, 24]. The averaging method employed in this study reduces the possibility of individual differences by using two dosimeters. The fact that the differences remained within ≤ 2% for OPFs ≤ 2 × 2 cm^2^ supports the effectiveness of this approach. The primary advantage of this approach lies in its capacity to evaluate dose outputs for small fields by combining facility-owned detectors with correction factors above and below unity, thereby offering a practical alternative to W2 PSD [15]. This makes the method particularly beneficial for RTPS modeling, commissioning, and routine quality assurance.

In the evaluation of the beam modeling approaches, the calculated OPFs between the M and RBD + M groups revealed minimal variations owing to using the same measurement OPFs. This suggests that the observed differences between the M and RBD + M groups are primarily owing to variations in the beam modeling process applied to the measured and RBD profile data [21]. At field sizes ≥ 2 × 2 cm^2^, incorporating measured OPFs for field sizes smaller than 3 × 3 cm^2^ in the M and RBD + M groups resulted in smaller deviations from the W2 PSD measurements compared with the RBD group. Furthermore, the standard deviation in the OPFs calculated across different beam energies was larger for the RBD group than for the M and RBD + M groups. These findings suggest that the differences in beam quality between this study’s data and the mean data from three TrueBeam linear accelerators used to acquire the RBD may limit modeling accuracy. However, this can be improved by incorporating the measured OPFs for field sizes ≥ 2 × 2 cm^2^.

The OPF differences in the RBD group tended to increase with larger irradiation diameters. This increase may be explained by slight deviations in field size, likely influenced by calibration deficiencies—in particular, the jaw position deviations and beam profile discrepancies between the RBD measurement facility and that used in this study [12, 25]. These results highlight the importance of comprehensive TPS commissioning, which should include the verification of small-field OPFs and the output of large irradiation fields. Moreover, the difference was larger for FF than for FFF, which may be owing to jaw position deviations with large tolerances between the RBD measurement facility and the facility used in this study.

Herein, the calculated OPF for the RBD group demonstrated the least variation for the 1 × 1 cm^2^ field, suggesting that RBD modeling alone can provide high accuracy for small-field output calculations. The significant difference in the calculated OPFs for the field size of 1 × 1 cm^2^ in the M and RBD + M groups may be influenced by the Eclipse-calculated collimator backscattering factors owing to a mismatch in incorporating OPF measurements for the ≥ 1 × 1 cm^2^ field size and scan profile data for the ≥ 2 × 2 cm^2^ field size in the RTPS [13]. The collimator backscattering factors calculated in Eclipse represent coefficients that quantify the difference between the OPFs generated by the TPS dose calculation algorithm and the registered OPF [13]. Accurate OPF registration may be achieved by verifying these factors and correcting or remodeling the OPF accordingly. This evaluation supports the feasibility of RBD modeling after setting the appropriate jaw position and beam quality, which offers the advantage of eliminating the need for direct measurements. In addition, it minimizes discrepancies arising from measurement errors caused by uncertainties such as chamber and measurement setting errors in small irradiation fields [25]. In this study, OPFs were assessed at an SSD of 90 cm and a depth of 10 cm, whereas the RBD data were obtained at an SSD of 95 cm and a depth of 5 cm. These differing measurement conditions may have contributed to minor OPF variations, likely owing to perturbations introduced by chamber and detector changes [12, 26].

When analyzing rectangular irradiation fields, the RBD group exhibited greater differences on the upper jaw side, whereas the M group showed larger discrepancies on the lower jaw side. This contrast may be attributed to variations in the detectors used for measurement, which can impact scattering and perturbation effects [27, 28]. Furthermore, the observed differences between the AAA and AXB algorithms for rectangular fields may be related to differences in model accuracy when estimating lateral scattering, which can be affected by geometric asymmetries between the upper and lower jaw configurations [29]. The upper jaw follows an arc trajectory that aligns with the photon beam path. Conversely, the lower jaw moves in a straight line perpendicular to the beam axis [30]. Consequently, differences in mechanical movements contribute to modeling accuracy variations. The observed decline in modeling accuracy for the lower jaw is likely attributable to these mechanical discrepancies. Therefore, we recommend that complex IMRT plans, particularly those employing multiple rectangular fields with one side ≤ 1 cm, be thoroughly verified using multiple types of dosimeters.

The uncertainties associated with the ionization chamber, EDGE diode, and W2 PSD are a limitation of this study. The measured OPFs are subject to Type-A standard deviations, which reflect the variability of repeated measurements, and Type-B uncertainties, arising from factors such as detector setup, collimator reproducibility in defining field size, linear accelerator stability, and temperature sensitivity [17]. Using multiple dosimeters for small irradiation fields has been reported to reduce uncertainty [31, 32]. This may be advantageous when using facility-owned detectors. In addition, certain uncertainties are specific to individual dosimeters—for example, the need to correct the Cherenkov light in the W2 PSD. As this study involved comparing W2 PSD measurements with beam modeling results derived from other dosimeters, these device-specific uncertainties likely contributed to the overall uncertainty profile of each dosimeter. However, this study used an ionization chamber and diode detector, which do not require correction for Cherenkov radiation and have a long history of widespread adoption, making them highly versatile. The averaging method presented in this study offers a practical approach that supports commissioning and quality assurance procedures across various facilities. Moreover, the study successfully demonstrated the utility of RBD by appropriate adjustment of the jaw and beam characteristics, highlighting its potential to streamline the implementation and validation of radiotherapy equipment in clinical practice.

## Conclusion

For small irradiation fields, the average OPFs obtained from the PinPoint 3D ionization chamber and EDGE diode were comparable to those measured with the W2 PSD. When using RBD-only modeling by adjusting the jaw position and beam characteristics, the calculated OPFs differed from the measured OPFs by less than 3.0% for field sizes ≤ 3 × 3 cm^2^, including rectangular fields. These findings demonstrate the feasibility of using averaging techniques for quality control and provide a valuable reference for institutions considering the adoption of RBD-only modeling in the commissioning and routine quality assurance of radiotherapy systems.
